# Leveraging PANoptosis-associated genes for unraveling implication of decidualization deficiency in pre-eclampsia via transcriptome data and experiment validation

**DOI:** 10.3389/fcell.2026.1677798

**Published:** 2026-03-04

**Authors:** Xiaoxuan Zhao, Yuanyuan Zhang, Qingnan Fan, Yang Zhao, Meiping Ding, Yiming Ma, Yan Yang, Aiwu Huang, Hongying Tang, Yuepeng Jiang, Hongli Zhao

**Affiliations:** 1 Department of Traditional Chinese Medicine (TCM) Gynecology, Hangzhou TCM Hospital Affiliated to Zhejiang Chinese Medical University, Hangzhou, China; 2 Research Institute of Women’s Reproductive Health, Zhejiang Chinese Medical University, Hangzhou, China; 3 The Third Clinical Medical College, Zhejiang Chinese Medical University, Hangzhou, China; 4 The Affiliated Hospital of Nanjing University of Chinese Medicine, Nanjing, China; 5 Wuyi County Hospital of Traditional Chinese Medicine, Jinhua, China; 6 Macau University of Science and Technology, Macau, China; 7 Hangzhou Lin’an Traditional Chinese Medicine Hospital, Hangzhou, China; 8 College of Basic Medical Science, Zhejiang Chinese Medical University, Hangzhou, China

**Keywords:** animal experiment, bioinformatics analysis, decidualization deficiency, machine learning, PANoptosis, pre-eclampsia

## Abstract

**Background:**

Decidualization deficiency is a key pathological feature of pre-eclampsia (PE) and is closely associated with aberrant regulation of cell fate. PANoptosis is a recently characterized form of inflammatory programmed cell death that has been implicated in several pregnancy-related disorders. However, its potential involvement in decidualization deficiency in PE remains poorly understood. This study aimed to explore the association between PANoptosis-related genes and decidualization deficiency in PE, and to identify candidate biomarkers and potential therapeutic targets related to PANoptosis.

**Methods:**

Datasets containing decidual tissue samples derived from women with PE and normal controls were acquired in the Gene Expression Omnibus (GEO) database. Differentially expressed genes (DEGs) were identified and subjected to enrichment analysis. After that, PANoptosis-related genes were intersected with DEGs derived from the decidual tissue of PE, followed by protein–protein interaction (PPI) network construction and correlation analysis. Next, the immune infiltration landscape and its association with PANoptosis-related DEGs were assessed. Furthermore, three machine learning algorithms, including support vector machine-recursive feature elimination (SVM-RFE), the least absolute shrinkage and selection operator (LASSO), and the random forest (RF) algorithms, were adopted to identify potential diagnostic biomarkers for PE. Artificial neural network (ANN) and nomogram models were then constructed and evaluated in testing datasets, which included decidual stromal cell samples derived from women with PE and normal controls. Additionally, the expression of PANoptosis-related signature genes and decidualization-related markers was experimentally validated in primary human decidual stromal cells (HDSCs) derived from PE patients and healthy controls. In addition, consensus clustering analysis was conducted on the basis of signature genes, and immune infiltration landscape analysis of different subtypes of PE was performed. Ultimately, the candidate compounds targeting the signature genes were screened and then further verified *in vivo* and *in vitro* models.

**Results:**

430 DEGs were determined, and enrichment analysis indicated that these DEGs were mainly involved in inflammation, apoptosis, and dysfunction of decidual tissue in PE. Then, 10 PANoptosis-related DEGs in PE were further screened. Following that, immune landscape analysis revealed an aberrant abundance of various immunocytes and the levels of immune checkpoints in the decidual tissue of PE, which were closely associated with the PANoptosis-related DEGs. Next, through machine learning, nine PANoptosis-related signature genes (*MAPK3*, *RIPK1*, *RIPK3*, *PYCARD*, *BAX*, *TUG1*, *CDK1*, *MAPK1*, and *TAB2*) were identified with favorable predictive performance. Besides, the ANN and nomogram models were constructed, and demonstrated high discriminative ability in the training dataset (AUC = 0.999, 95% CI: 0.995–1.000). Consistently, validation in primary HDSCs derived from PE patients and healthy controls confirmed dysregulated expression of these signature genes, accompanied by reduced decidualization markers (*PRL*, *IGFBP1*). Furthermore, on the basis of the analysis of nine signature genes, two different subtypes of PE were acquired, in which subtype B showed an immune hyperactivity state compared to subtype A. Furthermore, melatonin was identified as a candidate compound targeting PANoptosis-related genes and showed protective effects *in vivo* and *in vitro*, including improved blood pressure, reduced proteinuria, partial restoration of decidualization markers (PRL, IGFBP1, and F-actin), and declined expressions of PANoptosis-related signature genes (BAX, MAPK1, and MAPK3). Importantly, functional experiments demonstrated that MAPK3 knockdown markedly attenuated PANoptosis-associated inflammatory cytokine production, reduced BAX expression, and partially restored F-actin organization and decidualization markers under PANoptosis-inducing conditions.

**Conclusion:**

This study suggests a potential association between PANoptosis-related molecular dysregulation and decidualization deficiency in PE. The identified PANoptosis-related signature genes may serve as candidate biomarkers with predictive relevance, and melatonin may represent a potential therapeutic candidate targeting PANoptosis-related pathways. These findings provide a foundation for future mechanistic and translational studies on PE.

## Introduction

Pre-eclampsia (PE) is characterized by systolic blood pressure ≥140 mmHg and/or diastolic blood pressure ≥90 mmHg in previously normotensive women, accompanied by proteinuria and/or maternal organ dysfunction occurring after 20 weeks of gestation (Chappell et al., 2021). Approximately 8% of first pregnancies are affected by PE, resulting in at least 76,000 maternal fatalities and 500,000 newborn deaths globally (Ghulmiyyah and Sibai, 2012; Chappell et al., 2021). Currently, PE is considered a serious public health challenge, not only because of its acute, life-threatening risks to both mother and fetus, but also due to its long-term adverse consequences on maternal health, such as cardiovascular, renal, among others. Therefore, elucidating the underlying pathological mechanisms and identifying sensitive biomarkers and effective therapeutic targets for early diagnosis and intervention remain urgent priorities.

It is widely acknowledged that PE is associated with insufficient invasion of cytotrophoblasts into the maternal spiral arteries of the uterine decidua, leading to defective endovascular remodeling, impaired uteroplacental perfusion, and abnormal placentation. However, the mechanisms responsible for inadequate cytotrophoblast invasion remain unclear. Decidualization is a conceptus-independent process that involves the transformation of endometrial stromal cells (ESCs) into decidual stromal cells, accompanied by the recruitment of specialized immune cells and extensive vascular remodeling. This process is critical for controlling the depth and extent of trophoblast invasion (Gellersen and Brosens, 2014). Recent findings revealed that defective decidualization is a key contributor to the development of PE (Garrido-Gomez et al., 2021; Ma et al., 2021; Garrido-Gómez et al., 2022). Such defects are associated with reduced resistance to oxidative stress, excessive inflammation, and dysregulated immune tolerance, thereby compromising trophoblast invasion (Yang et al., 2018). Despite growing insights into molecular alterations associated with deficient decidualization in pre-eclampsia, the integrated regulatory mechanisms underlying this process remain incompletely understood (Sa, 2016; Deer et al., 2023).

PANoptosis is a coordinated form of programmed cell death mediated by the PANoptosome complex, enabling extensive crosstalk among pyroptosis, apoptosis, and necroptosis (Sun et al., 2024). To date, three distinct PANoptosome complexes have been identified, including ZBP1-, AIM2-, and RIPK1-PANoptosomes (Gullett et al., 2022). Accumulating evidence suggested a close association between PANoptosis and PE. Baev et al. reported that ZBP1 was highly expressed in villous syncytiotrophoblasts in early-onset PE and was associated with impaired placental morphofunction (Baev et al., 2019). Furthermore, Li et al. and Hannan et al. demonstrated that AIM2 and RIPK1 activation promoted the generation of pro-inflammatory and antiangiogenic substances, correlating with disease severity (Li et al., 2020; Samir et al., 2020). In addition, defective decidualization has been linked to individual forms of cell death, including pyroptosis, apoptosis, and necrosis (Akaeda et al., 2021; Zhu et al., 2021). However, the potential involvement of PANoptosis in impaired decidualization in the context of PE is still poorly explored.

**FIGURE 1:**
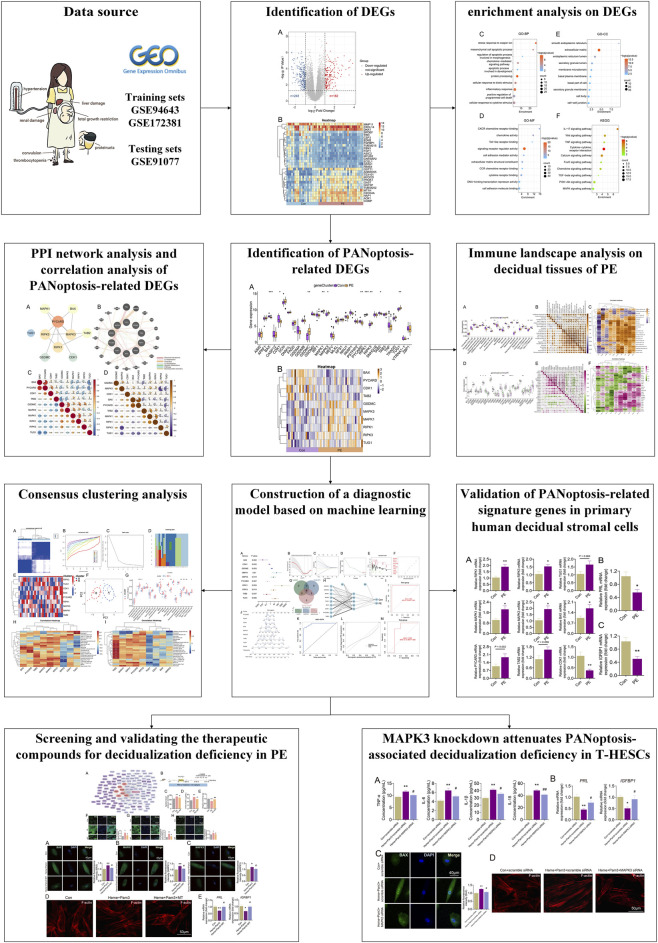
Flow chart of leveraging PANoptosis-associated genes for unraveling implication of decidualization deficiency in pre-eclampsia.

In this study, we utilized integrative bioinformatics analyses combined with machine-learning approaches, as well as *in vivo* and *in vitro* experiments, to investigate the pathogenic mechanisms, candidate biomarkers, and potential compounds for PE from a PANoptosis-related perspective. The flowchart is displayed in Figure 1. This work may offer a new perspective on the pathogenesis of decidualization deficiency in PE on the basis of PANoptosis and provide a basis for diagnostic strategies and potential therapeutic intervention for PE.

## Materials and methods

### Datasets collecting and processing

All appropriate datasets were sourced from the Gene Expression Omnibus (GEO) database (https://www.ncbi.nlm.nih.gov/geo/) (Edgar et al., 2002) in the study. The keywords included “preeclampsia,” “pregnancy hypertension,” “hypertensive disorders of pregnancy,” “pregnancy-induced hypertension,” and “gestational hypertension”. And the datasets were further selected on the basis of criteria such as sequencing type (transcriptology), animal species (*Homo sapiens*), and sample source (decidual tissues or decidualized human endometrial stromal cells). Finally, three gene expression datasets were identified, comprising two datasets with 48 decidual tissue samples (GSE94643 and GSE172381) and one dataset consisting of 10 samples of decidualized human endometrial stromal cells (GSE91077). Among them, decidual tissue transcriptomes were used for the comprehensive analysis of the link between PANoptosis and PE, while the cell samples were employed to validate the diagnostic performance of the PANoptosis-related DEGs. The detailed information of each dataset was available in Table 1. The raw microarray data sets were processed and analyzed separately. The R “affy” package was carried out to perform the RMA normalization, including background correction, log_2_ transformation, quantile normalization, and probe summarization.

**TABLE 1:** The basic information of included dataset.

GSE no.	No. of samples	Platform	Description	Country
GSE94643	4 vs. 4	Affymetrix human gene 2.0 ST array	Decidua basalis from 4 PE patients and 4 normal controls	United States
GSE172381	16 vs. 24	Illumina NextSeq 500 (*Homo sapiens*)	Decidua from 24 PE patients and 16 normal controls	Spain
GSE91077	5 vs. 5	Agilent-026652 whole human genome microarray 4 × 44K v2	Decidualized human endometrial stromal cells from 5 PE patients and 5 normal controls	Spain

### Differentially expressed genes in PE

Differentially expressed genes (DEGs) were genes with a statistically significant difference at the transcription level. The gene expression matrices were prepared by the Perl (Strawberry Edition). Afterwards, DEGs were identified employing the R “limma” package from the long-expression value. The cut-off criteria of *P*-value < 0.05 and |logFC| > 1 were set to screen DEGs in PE with the limma package. Ultimately, DEGs were shown in volcano plots and heatmaps with the R “ggplot2” package.

### Functional annotation and pathway enrichment analysis on genes of the DEGs

The Gene Ontology (GO) enrichment analysis provided organized, computable data concerning the biological process (BP), molecular function (MF), and cellular component (CC) of genes. The Kyoto Encyclopedia of Genes and Genomes (KEGG) enrichment analysis was a widely used method for systematically exploring gene functions and gene pathways. To functionally annotate genes of the DEGs identified by the aforementioned comparison groups, annotation and visualization of GO and KEGG enrichment analyses were conducted on the basis of Metascape (http://metascape.org/) (Zhou et al., 2019). *P-*value < 0.05 was deemed to meet the criteria.

### Identification of PANoptosis-related DEGs, protein–protein interaction network analysis and correlation analysis

Sixty-three PANoptosis-related genes were gathered from GeneCards (https://www.genecards.org/) (Safran et al., 2010) and the previously published studies (Christgen et al., 2020; Wang and Kanneganti, 2021). Genes differentially expressed between PE and control samples were designated as PANoptosis-related DEGs. In order to understand the functions that these genes may perform, information on tissue localization and molecular function was downloaded from the BioGPS (http://biogps.org) (Wu et al., 2016), the Human Protein Atlas (https://www.proteinatlas.org/) (Pontén et al., 2011), GeneCards (https://www.genecards.org/) (Safran et al., 2010), Alliance of Genome Resources (https://www.alliancegenome.org/) (Alliance of Genome Resources Consortium, 2020), and UniProt (https://www.uniprot.org/) (UniProt Consortium, 2021), respectively. Then, to gain insight into the molecular biochemical response network, the PANoptosis-related DEGs of PE were imported into the STRING database (version 11.5; www.string-db.org) (Szklarczyk et al., 2023) to establish the protein-protein interaction (PPI) network, with the least desired interaction score set at 0.400. And then the PPI network was visualized by the R “corrplot” package. Besides, the Cytoscape plug-in CytoHubba was used to select hub genes (Chin et al., 2014), in which genes with MCC algorithms ≥3 were chosen as the hub genes. Furthermore, we also analyzed the networks of the PANoptosis-related DEGs through GeneMANIA (http://genemania.org) (Franz et al., 2018). After that, we installed and loaded the corrplot package, then prepared the data, and used the cor() function to calculate the correlation coefficients between variables in the data frame. Next, we set the method parameter to “spearman” to specify the use of Spearman’s correlation coefficients. Finally, the correlation analysis was utilized to examine the relationship between PANoptosis-related DEGs by using the Spearman correlation coefficient. The “corrplot” package in R software was utilized for visualization.

### Immune infiltration landscape estimation

Single-sample gene-set enrichment analysis (ssGSEA) is an extension of the GSEA method, which allows defining an enrichment fraction to represent the absolute enrichment degree of a gene set in each sample in a given data series (Bindea et al., 2013). At this time, ssGSEA was performed by using the R package “GSVA” to explore the different infiltration degrees of immunocytes in the PE and healthy controls. Besides, the Mann-Whitney test was carried out to compare the expression profiles of the immune checkpoints in decidual tissue between PE patients and healthy controls. In addition, the R “clusterProfiler” program was applied to conduct the correlation analysis between the PANoptosis-related DEGs and immunocytes and immune checkpoints between groups. *P*-value < 0.05 was regarded as a significant difference.

### Screening candidate diagnostic biomarkers based on machine learning

The machine learning algorithms were applied to evaluate candidate biomarkers for predicting PE, including the binary logistic regression (BLR), the support vector machine-recursive feature elimination (SVM-RFE), the least absolute shrinkage and selection operator (LASSO) and the random forest (RF) algorithms. Firstly, BLR was performed to identify signature genes from PANoptosis-related DEGs in the R “rms” package. SVM-RFE is able to accurately derive a subset of genes based on classification and regression, which can be used to find the optimal variables. The R “kernlab,” “e1071” and “caret” packages were adopted to implement SVM-RFE classification of the selected biomarkers of PE. LASSO is a regression algorithm that applies regularization to select variables and enhances the predictive precision and understandability of a statistical model. To conduct the LASSO algorithm, the R “glmnet” package was utilized with alpha = 1. RF is an appropriate algorithm to predict continuous variables and build a classifier in the context of high-dimensional data, with the benefits of achieving a high degree of predictive accuracy, sensitivity, and specificity. To conduct LASSO algorithm, the R “glmnet” package was utilized with alpha = 1. RF is an appropriate algorithm to predict continuous variables and build a classifier in the context of high-dimensional data, with the benefits of achieving a high degree of predictive accuracy, sensitivity, and specificity. Here we utilized the R “randomForest” package to perform RF. To evaluate model robustness and generalizability, 10-fold cross-validation was performed, in which the dataset was randomly divided into ten subsets, with nine used for training and one for validation in each iteration. Average performance across all folds was calculated. The overlapped genes of the three algorithms in the Venn diagrams were considered candidate signature genes in PE diagnosis. Additionally, the R “neuralnet” and “neuralnettools” packages were employed to construct the artificial neural network (ANN) of the signature genes based on their gene scores. And the receiver operating characteristic (ROC) curve was established by employing the R “pROC” package, and the calculation of area under the ROC (AUC) was performed. Finally, on the basis of the candidate genes, the “rms” R package was developed to establish the nomogram prediction model, in which “points” represents the score of candidate genes and “total points” represents the sum of all the scores of the genes aforementioned. Besides, an ROC curve was performed to determine whether the decision based on the nomogram benefited the diagnosis of PE. And the calibration curves were constructed to evaluate the predictive efficiency of the nomogram in PE. In addition, the predicted efficacy of the signature genes was verified in datasets of GSE91077 that contained the decidualized human endometrial stromal cells from patients with PE and healthy controls. Additionally, a ROC curve analysis was also conducted to validate the diagnostic accuracy of the ANN model in the dataset of GSE91077.

### Consensus clustering analysis and immune infiltration landscape analysis of different subtypes

In order to explore the gene expression profiles of the signature genes in different PE populations, the R “ConsensusClusterPlus” package was utilized to conduct unsupervised cluster analysis. The criteria of classification were set to a maximum number of 9, replicates of 50, a sample share of 0.8, and a feature-to-sample ratio of 1, which was replicated 1,000 times to ensure the reliability of the results. The ideal cluster numbers were identified by the consensus cumulative distribution function (CDF) plot. The principal component analysis (PCA) was utilized to define the expression differences of signature genes in different subtypes using the R “ggplot2” package. Besides, the expression of immunocytes in distinct subtypes was quantified by the R “GSVA” package and displayed in box plots, as mentioned before. To further figure out the contribution of signature genes to the immune infiltration landscape of subtypes, the association of signature genes with infiltrated immunocytes was explored using Spearman correlation analysis.

### Screening potential drugs for PE by targeting the PANoptosis-related signature genes

Potential compounds that were capable of targeting the signature genes were screened through the databases listed below: the Comparative Toxicogenomics Database (CTD) (http://ctdbase.org/) (Davis et al., 2023) and the DrugBank (https://go.drugbank.com/) (Wishart et al., 2018). Compounds that satisfy the below criteria will be incorporated: (1) drug-gene pairs with interaction count ≥9; (2) drugs that are FDA-approved. Furthermore, the association between the medication and the target was displayed through Cytoscape software (3.7.1).

### Experimental validation of PANoptosis-related signature genes and therapeutic targeting in pre-eclampsia

#### Human decidual tissue collection

Human decidual tissues were collected aseptically during cesarean section from women diagnosed with pre-eclampsia (PE, n = 6) and gestational age–matched uncomplicated pregnant women (healthy controls, n = 6) at Wuyi County Hospital of Traditional Chinese Medicine. PE was diagnosed according to standard criteria (ACOG Committee on Practice Bulletins--Obstetrics, 2002) as new-onset hypertension (systolic blood pressure ≥140 mmHg or diastolic blood pressure ≥90 mmHg) after 20 weeks of gestation in a previously normotensive woman, accompanied by proteinuria (≥0.3 g protein in a 24-h urine collection). Inclusion criteria comprised intrauterine singleton pregnancy, maternal age ≥18 years, informed consent, and fulfillment of the diagnostic criteria for either PE or uncomplicated pregnancy. Exclusion criteria included chronic hypertension, gestational hypertension without preeclampsia, diabetes mellitus, autoimmune diseases, placental abnormalities (e.g., hydatidiform mole), or other severe maternal complications (Gumilar et al., 2025). Decidual tissues were carefully dissected from the maternal side of the placenta within 30 min after delivery, immediately snap-frozen in liquid nitrogen, or fixed for subsequent molecular and histological analyses. This study was approved by the Ethics Committee of Wuyi County Hospital of Traditional Chinese Medicine (Approval No. 2025-LL-007), and written informed consent was obtained from all participants prior to surgery.

#### Isolation and cultivation of primary human decidual stromal cells

After separation of decidual tissues from the placenta, samples were rinsed thoroughly with sterile phosphate-buffered saline (PBS) to remove blood and debris. The tissues were then finely minced and digested with 0.1% collagenase type I (Sigma-Aldrich, United States) and 0.01% DNase I (Sigma-Aldrich, United States) in DMEM/F12 at 37 °C for 45 min with gentle agitation. The resulting cell suspension was filtered through a 70 μm cell strainer and centrifuged at 300 *g* for 5 min. The pellet was resuspended in phenol red-free DMEM/F12 supplemented with 10% charcoal/dextran-treated FBS, 100 U/mL penicillin, and 100 μg/mL streptomycin, then seeded in culture plates. Non-adherent cells were removed after 24 h, and adherent decidual stromal cells were cultured at 37 °C under 5% CO_2_.

#### Animals and administration schedule

C57BL mice (aged 6–8 weeks), obtained from the Experimental Animal Center of Zhejiang Chinese Medical University (SYXK(Z) 2021-0012), were provided with *ad libitum* access to food and water and raised in a ventilated setting with a 12:12 h light-dark cycle. After acclimatization feeding, female and male mice were mated in a proportion of 2:1. The copulation plug was checked regularly on a daily basis, and the day the copulation plug was first detected was identified as the first day of gestation (E0.5). Pregnant mice were randomized into control, PE and PE + melatonin groups, with six mice in each group. The L-NAME–induced PE mice model was used in this study. N(omega)-nitro-L-arginine methyl ester (L-NAME) decreases the vasodilator effect of nitric oxide (NO) and induces PE in mice (Motta et al., 2015). Mice in the PE group were subcutaneously injected with L-NAME (125 mg/kg/day, Cat. HY-18729A, MedChemExpress) at E10.5 and lasted for 8 days. At the same time, the melatonin group was administered melatonin (10 mg/kg/day, Cat. HY-B0075, MedChemExpress) based on the L-NAME-induced PE model. The control group and the L-NAME group were treated with the same volume of saline. The non-invasive animal blood pressure monitor was employed to measure the systolic blood pressure (SBP) on the day of E16.5 in every case, specifically between 9 and 11 a.m. Urine samples were taken on E16.5 to measure the concentration of urinary proteins. Pregnant mice were administered pentobarbital anesthesia on E17.5. The decidual tissues were collected after anesthesia. All experimental protocols were conducted in compliance with institutional guidelines and authorized by the Animal Experimental Committee of Zhejiang Chinese Medical University (NO. IACUC-20241021-05).

#### Cell culture and *in vitro* decidualization

Consistent with previous literature (Huang et al., 2017), the human endometrial stromal cells (T-hESCs, ATCC) were cultured in phenol red-free DMEM/F12 (Gibco, United States) supplemented with 10% charcoal/dextran-treated FBS (Gibco, United States), 100 U/mL penicillin (Beyotime, China), and 100 μg/mL streptomycin (Beyotime, China) at 37 °C under 5% CO_2_ humidified air. To induce decidualization *in vitro*, T-HESCs were treated with 0.5 mM 8-bromo-cAMP (MedChemExpress, United States) and 1 μM medroxyprogesterone acetate (MPA) (MedChemExpress, United States) for 6 days. The medium was renewed every 48 h. The decidualization of T-hESCs was evaluated by observing the morphological phenotype using immunofluorescence and quantifying the IGFBP1 and PRL expressions using quantitative reverse-transcription PCR (qRT-PCR).

#### Cell processing and grouping

T-hESCs were randomly allocated into three groups, including control group, PANoptosis group, and melatonin group. The control group was treated with 0.5 mM 8-bromo-cAMP and 1 μM MPA for 6 days to induce decidualization. In the PANoptosis group, cells were treated with 50 μM Hemin (heme, H9039, Sigma) in combination with 500 ng/mL Pam3CSK4 (Pam3, tlrl-pms, InvivoGen) on the 4.5 days of decidualization for 36 h to induce PANoptosis (Sundaram et al., 2024). The melatonin group was treated with 10 nM melatonin on the fifth day of decidualization for 24 h after PANoptosis stimulation by heme and Pam3.

#### siRNA transfection and functional validation

To verify the functional role of the identified hub gene MAPK3 in decidualization deficiency, small interfering RNA (siRNA) targeting MAPK3 (MAPK3 siRNA) and a negative control scramble siRNA were synthesized. T-hESCs were transfected with MAPK3 siRNA or scramble siRNA using Lipofectamine 3000 (Invitrogen, United States) according to the manufacturer’s instructions. Primer sequences were as followed: MAPK3 siRNA1:CAACATGAAGGCCCGAAACTA, MAPK3 siRNA2:GCAGCTGAGCAATGACCATAT, MAPK3 siRNA3:CCTGAATTGTATCATC AACAT. In this study, siRNA3 exhibited the most significant effect on the downregulation of MAPK3 compared with other interference sequences (Supplementary Figure S1). Following treatment, qRT-PCR analysis of decidualization markers, including IGFBP1 and PRL, was conducted. In parallel, culture supernatants were collected for enzyme-linked immunosorbent assay (ELISA) to measure the concentrations of inflammatory cytokines IL-1β, IL-6, TNF-α, and IL-18. In addition, the decidualization of the morphological phenotype and PANoptosis-related signature gene BAX expression was examined by immunofluorescence staining.

#### Enzyme-linked immunosorbent assay (ELISA)

Urine samples from pregnant mice and cell culture supernatants from T-hESCs were collected and centrifuged at 800 *g* for 5 min to remove debris. Albumin concentrations were detected using a mouse albumin ELISA kit (Cat. MU30662, Bioswamp) according to the manufacturer’s protocol. For *in vitro* experiments, the concentrations of inflammatory cytokines, including IL-1β, IL-6, TNF-α, and IL-18, in T-hESC culture supernatants were determined using commercially available human ELISA kits (IL-1β, Cat. BY-EH110351; IL-6, Cat. BY-EH110377; TNF-α, Cat. BY-EH111776; IL-18, Cat. BY-EH110349, BYabscience) following the manufacturers’ protocols. Optical densities were measured at 450 nm using a microplate reader, and analyte concentrations were calculated based on standard curves.

#### Quantitative real-time polymerase chain reaction (qRT-PCR)

After sufficient grinding, total RNA was extracted from primary human decidual stromal cells (HDSCs) and T-hESCs using the TRIZOL reagent, respectively. Then the HiFiScript cDNA synthesis kit (Cat. CW2569M, CWBIO) was utilized for reverse transcription. The SYBR Green Pro Taq HS premixed qPCR kit (Cat. AG11701, AGBIO) was also employed for the PCR system. The 2^−△△CT^ method was adopted to calculate relative gene expression. In primary HDSCs, qRT-PCR was used to assess the mRNA expression of decidualization markers (PRL and IGFBP1) and PANoptosis-related signature genes, including MAPK3, RIPK1, RIPK3, PYCARD, BAX, TUG1, CDK1, MAPK1, and TAB2. In T-hESCs, the mRNA expression levels of decidualization markers PRL and IGFBP1 were evaluated by qRT-PCR following the indicated treatments. Primer sequences were provided in Supplementary Table S1.

#### Immunofluorescence staining

Frozen sections of mouse decidual tissues, and T-hESCs slides were prepared. The slides were fixed in 4% paraformaldehyde solution for 30 min. Then, the sections were washed and blocked. For mouse decidual tissues and T-hESCs, primary antibodies were applied overnight at 4 °C as follows: BAX (1:200, Cat. 48690, Signalway Antibody), MAPK1 (1:200, Cat. ET1603-23, HUABIO), and MAPK3 (1:200, Cat. ET1604-16, HUABIO). After being washed three times, the sections were followed by secondary antibodies at room temperature in the dark for 2 h: goat anti-rabbit IgG H&L (Alexa Fluor® 488) (1:200, Cat. No: ab150077, Abcam). To visualize F-actin for morphological analysis, sections of T-hESCs were incubated with phalloidin (Cat. PF00003, Proteintech) for 20 min. The sections were stained with DAPI. Imaging was performed using a digital pathological section (fluorescence) scanning analyzer (VS120-S6-W, OLYMPUS) for mouse decidual tissues, and a laser confocal microscope (LSM880, Zeiss) for T-hESCs. The mean immunofluorescence intensity capture and analysis of BAX, MAPK1, and MAPK3 were performed by ImageJ software (National Institutes of Health, Bethesda, Maryland, https://imagej.net/ij/disclaimer.html).

### Statistical analysis

The statistical analyses were conducted using R software version 4.3.0 and GraphPad Prism Version 9.4.0 (GraphPad Software, San Diego, CA, United States). Specifically, the R packages “kernlab,” “e1071,” “glmnet,” “randomForest,” and “ConsensusClusterPlus” follow the GPL version 2 license (http://gnu.org/licenses/gpl2.html), while “limma,” “GSVA,” “rms,” “neuralnet,” and “pROC” follow the GPL version 3 license (https://www.gnu.org/licenses/gpl-3.0.html). The R packages “ggplot2,” “corrplot,” and “caret” are licensed under the MIT License (https://opensource.org/licenses/MIT). The R package “clusterProfiler” is licensed under the Artistic-2.0 license (https://opensource.org/licenses/Artistic-2.0). And the “neuralnettools” package is licensed under CC0 (https://creativecommons.org/publicdomain/cc0/). The Student’s t-test was employed to compare continuous variables between two groups (Kalpić et al., 2011). We performed the paired t-test using the statistical software R (Krzywinski and Altman, 2014). In R, we used the t.test() function and set paired = TRUE to specify that we were conducting a paired t-test. They are all open-source licenses that encourage the sharing and collaborative development of software. One-way ANOVA was applied to assess the indicators among groups (Janczyk and Pfister, 2023), followed by Dunnett’s test for *post hoc* comparisons, which controls the family-wise error rate for multiple comparisons against a single control group (Tallarida and Murray, 1987). A *P-*value < 0.05 indicated statistical significance.

## Results

### Identification of DEGs and enrichment analysis

**FIGURE 2:**
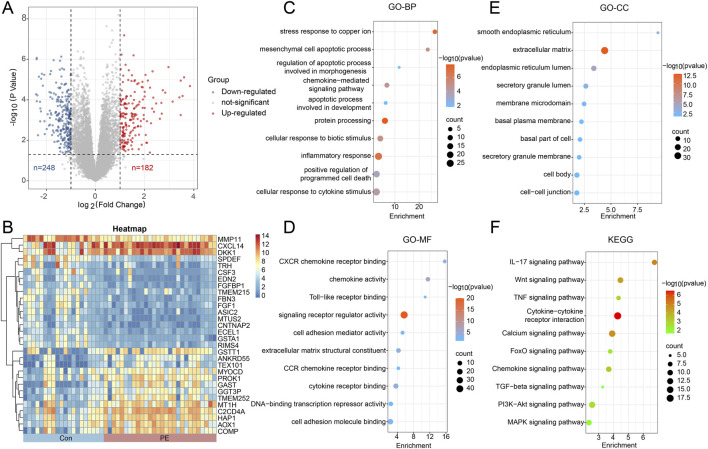
Identification and enrichment analysis of DEGs. **(A)** The volcano plot depicts all DEGs in the decidual tissues between PE and normal controls, of which red points represent upregulated DEGs and blue points represent downregulated DEGs. **(B)** The heatmaps of DEGs presented by the decidual tissue samples of PE cases or controls (X-axis) and DEGs (Y-axis). **(C–E)** The GO analysis of DEGs, including biological process, cellular component and molecular function respectively and **(F)** the KEGG analysis of DEGs from the decidual tissues between PE and normal controls. The X-axis represents enrichment and the Y-axis refers to GO or KEGG terms. The circle size represents counts and the color refers to *P*-value.

As shown in Figures 2A,B, 430 DEGs in the decidual tissues of PE were obtained by the limma package in R software, consisting of 182 upregulated genes and 248 downregulated genes (*P* < 0.05). Then the DEGs underwent further processing for functional enrichment by GO and KEGG pathway analyses. In the GO-BP category, DEGs were mainly enriched in stress response to copper ion, mesenchymal cell apoptotic process, and regulation of apoptotic process involved in morphogenesis. In the GO-CC category, DEGs were related to the smooth endoplasmic reticulum, extracellular matrix, and membrane microdomain. In the GO-MF category, DEGs were significantly correlated with CXCR chemokine receptor binding, toll-like receptor binding, and signaling receptor regulator activity. Similarly, KEGG analysis demonstrated that DEGs were mainly involved in the IL-17 signaling pathway, the Wnt signaling pathway, and the TNF signaling pathway (Figures 2C–F). These enrichment results indicated a strong association between PE and dysregulated inflammatory pathways, accompanied by changes in cell function and fate.

### PANoptosis-related DEGs identification and tissue localization

**FIGURE 3:**
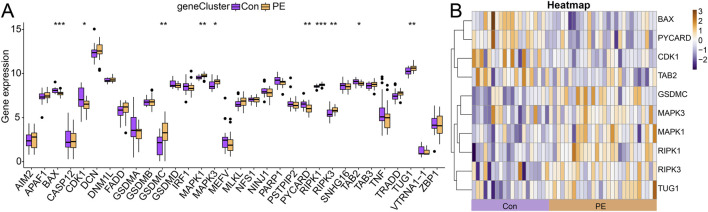
Identification of PANoptosis-related DEGs. **(A)** The boxplots depict different gene expressions of PANoptosis-related DEGs in the decidual tissues between PE and normal controls. * represents *P* < 0.05, ** represents *P* < 0.01 and *** represents *P* < 0.001 compared with normal controls. **(B)** The heatmaps of PANoptosis-related DEGs in the decidual tissues between PE and normal controls.

Due to the involvement of different forms of apoptosis in the pathogenesis of PE revealed by the enrichment analysis of the DEGs, we further investigated the critical function of PANoptosis-related genes in the decidual tissue of PE. Subsequently, a total of 63 PANoptosis-related genes was determined according to the database and previous studies (Christgen et al., 2020; Wang and Kanneganti, 2021). The details were listed in Supplementary Table S2. Among these, 10 PANoptosis-related genes were differentially expressed between PE and control samples, including six upregulated and four downregulated genes (Figures 3A,B). Comprehensive information on PANoptosis-related DEGs of PE was obtained from the BioGPS database, GeneCards, the Alliance of Genome Resources and the UniProt website. The results demonstrated that PANoptosis-related DEGs could be expressed in the endometrium tissue and immunocytes, suggesting that decidual tissue dysfunction in PE was associated with immune dysregulation and inflammation, and that PANoptosis-related DEGs may be involved in aberrant immune responses in PE (Table 2).

**TABLE 2:** Comprehensive information on PANoptosis-related DEGs.

Gene	Description (referring to the genecards database)	Expression in endometrium tissue (referring to the human protein Atlas database)	Expression in immocytes (referring to the bioGPS database)	Subcellular summary (referring to the human protein Atlas database)	Function (referring to the bioGPS, genecards, Alliance of Genome Resources and Uniprot database)	The localization of differentially expressed genes
GSDMC	Gasdermin C	YES	CD14+ Monocyte, CD4+ T cell, Native B cell, Plasma cell	Vesicles	GSDMC is a pore-forming protein that causes membrane permeabilization and pyroptosis (PMID:27281216, PMID:32929201, PMID:34012073). It is produced by the cleavage of gasdermin-D by caspase CASP8 in response to death signals (PMID:32929201, PMID:34012073). Homooligomerizes within the membrane and forms pores of 10–15 nm (nm) of inner diameter, triggering pyroptosis (PMID:32929201, PMID:34012073)	Decidual tissue
RIPK1	Receptor interacting serine/Threonine kinase 1	YES	CD14+ Monocyte, CD3+ T cell, CD4^+^CD25+ high regulatory T cell, CD4+ T cell, lymphocytes, memory B cell, monocyte, Native B cell, Native CD4+ T cell, neutrophil, Plasma cell, T cell	Plasma membrane and Cytosol	Serine-threonine kinase which is a key regulator of TNF-mediated apoptosis, necroptosis and inflammatory pathways (PMID:32657447, PMID:31827280, PMID:31827281) RIPK1 exhibits kinase activity-dependent functions that regulate cell death and kinase-independent scaffold functions regulating inflammatory signaling and cell survival (PMID:11101870, PMID:19524512)	Decidual tissue
TUG1	Taurine upregulated 1	YES	CD14+ Monocyte, CD3+ T cell, CD4^+^CD25+ high regulatory T cell, CD4+ T cell, lymphocytes, memory B cell, monocyte, Native B cell, Native CD4+ T cell, neutrophil, Plasma cell, T cell	-	TUG1 is predicted to enable cis-regulatory region sequence-specific DNA binding activity and act upstream of or within several processes, including mitochondrion organization, photoreceptor cell development, and spermatogenesis (provided by Alliance of Genome Resources, April 2022)	Decidual tissue
MAPK1	Mitogen-activated protein kinase 1	YES	CD14+ Monocyte, CD3+ T cell, CD4^+^CD25+ high regulatory T cell, CD4+ T cell, lymphocytes, memory B cell, monocyte, Native B cell, Native CD4+ T cell, neutrophil, Plasma cell, T cell	Cytosol and Nuclear speckles	The protein encoded by MAPK1 is a member of the MAP kinase family. MAP kinases, also known as extracellular signal-regulated kinases (ERKs), act as an integration point for multiple biochemical signals, and are involved in a wide variety of cellular processes such as proliferation, differentiation, transcription regulation and development (PMID:16393692)	Decidual tissue
RIPK3	Receptor interacting serine/Threonine kinase 3	YES	CD14+ Monocyte, CD3+ T cell, CD4^+^CD25+ high regulatory T cell, CD4+ T cell, lymphocytes, memory B cell, monocyte, Native B cell, Native CD4+ T cell, neutrophil, Plasma cell, T cell	-	Necroptosis, a programmed cell death process in response to death-inducing TNF-alpha family members, is triggered by RIPK3 following activation by ZBP1 (PMID:19524512, PMID:19524513) Activated RIPK3 forms a necrosis-inducing complex and mediates phosphorylation of MLKL, promoting MLKL localization to the plasma membrane and execution of programmed necrosis characterized by calcium influx and plasma membrane damage (PMID:19524512, PMID:19524513, PMID:22265413)	Decidual tissue
MAPK3	Mitogen-activated protein kinase 3	YES	CD14+ Monocyte, CD3+ T cell, lymphocytes, memory B cell, monocyte, Native B cell, Native CD4+ T cell, neutrophil, Plasma cell, T cell	Nucleoplasm	The substrates of MAPK3 include regulators of apoptosis, such as BAD, BTG2, CASP9, DAPK1, IER3, MCL1 and PPARG, all of which are responsible for the processes of apoptosis (PMID:35216969)	Decidual tissue
BAX	BCL2 associated X, apoptosis regulator	YES	CD14+ Monocyte, CD3+ T cell, CD4^+^CD25+ high regulatory T cell, CD4+ T cell, lymphocytes, monocyte, Native B cell, Native CD4+ T cell, neutrophil, Plasma cell, T cell	-	BAX plays a role in the mitochondrial apoptotic process (PMID:10772918, PMID:16113678). Under stress conditions, BAX undergoes a conformation change that causes translocation to the mitochondrion membrane, leading to the release of cytochrome c that then triggers apoptosis (PMID:11060313, PMID:16199525). It promotes activation of CASP3, and thereby apoptosis (PMID:11060313)	Decidual tissue
PYCARD	PYD and CARD Domain containing	YES	CD14+ Monocyte, CD3+ T cell, CD4^+^CD25+ high regulatory T cell, CD4+ T cell, lymphocytes, memory B cell, monocyte, Native B cell, Native CD4+ T cell, neutrophil, Plasma cell, T cell	Nucleoplasm, Nucleoli, and Cytosol	PYCARD functions as key mediator in apoptosis and inflammation (PMID:17599095) and it promotes caspase-mediated apoptosis involving predominantly caspase-8 and also caspase-9 in a probable cell type-specific manner (PMID:11103777) It mediates caspase-1-dependent inflammation that leads to macrophage pyroptosis, a form of cell death (PMID:24630722)	Decidual tissue
CDK1	Cyclin dependent kinase 1	YES	CD4^+^CD25+ high regulatory T cell, memory B cell, Plasma cell	Nucleoplasm and Cytosol	In proliferating cells, CDK1-mediated FOXO1 phosphorylation at the G2-M phase represses FOXO1 interaction with 14-3-3 proteins and thereby promotes FOXO1 nuclear accumulation and transcription factor activity, leading to cell death of postmitotic neurons (PMID:18356527) And it is essential for early stages of embryonic development (PMID:18480403, PMID:20360007)	Decidual tissue
TAB2	TGF-beta activated kinase 1 (MAP3K7) binding protein 2	YES	CD14+ Monocyte, CD3+ T cell, CD4^+^CD25+ high regulatory T cell, CD4+ T cell, lymphocytes, memory B cell, monocyte, Native B cell, Native CD4+ T cell, neutrophil, Plasma cell, T cell	-	The RanBP2-type zinc finger (NZF) of TAB2 specifically recognizes Lys-63′-linked polyubiquitin chains unanchored or anchored to the substrate proteins such as RIPK1/RIP1 and RIPK2: this acts as a scaffold to organize a large signaling complex to promote autophosphorylation of MAP3K7/TAK1, and subsequent activation of I-kappa-B-kinase (IKK) core complex by MAP3K7/TAK1 (PMID:15327770, PMID:18079694, PMID:22158122)	Decidual tissue

### PPI network analysis and correlation analysis of PANoptosis-related DEGs

**FIGURE 4:**
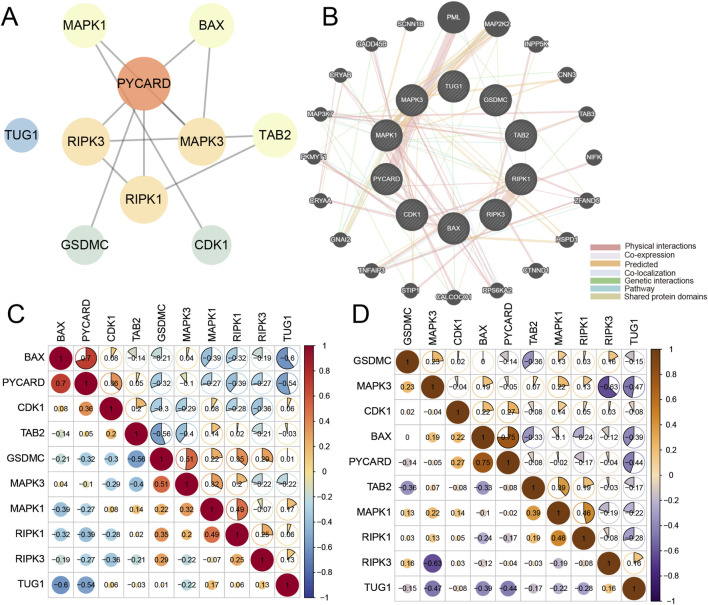
The PPI network of PANoptosis-related DEGs and correlation analysis of these genes in all samples and PE samples. **(A)** The PPI network is composed of PANoptosis-related DEGs from the decidual tissues between PE and normal controls in which PYCARD, RIPK3, MAPK3, and PIPK1 were regarded as hub genes. The circle nodes represent hub genes and edges refer to the interactions between nodes. **(B)** The networks of the PANoptosis-related DEGs in the decidual tissues between PE and normal controls were predicted by the GeneMANIA. **(C, D)** The correlation heatmaps reveal the relationship between PANoptosis-related DEGs in all the decidual samples and in PE decidual samples.

The PPI network of PANoptosis-related DEGs confirmed that these DEGs were closely correlated with each other. Four hub genes were obtained through the PPI network, including PYCARD, RIPK3, MAPK3 and PIPK1 (Figure 4A). Moreover, we also analyzed the networks of the PANoptosis-related DEGs through GeneMANIA, and the result suggested that these genes were closely linked both in localization and functions (Figure 4B). Correlation analysis showed that, across all samples, BAX-PYCARD (r = 0.70) and BAX-TUG1 (r = −0.60) represented the strongest positive and negative correlations, respectively (Figure 4C). In PE samples specifically, BAX was most positively correlated with PYCARD (r = 0.75), whereas RIPK3 exhibited the strongest negative correlation with MAPK3 (r = −0.63) (Figure 4D).

### Immune landscape analysis on the decidual tissues of PE

**FIGURE 5:**
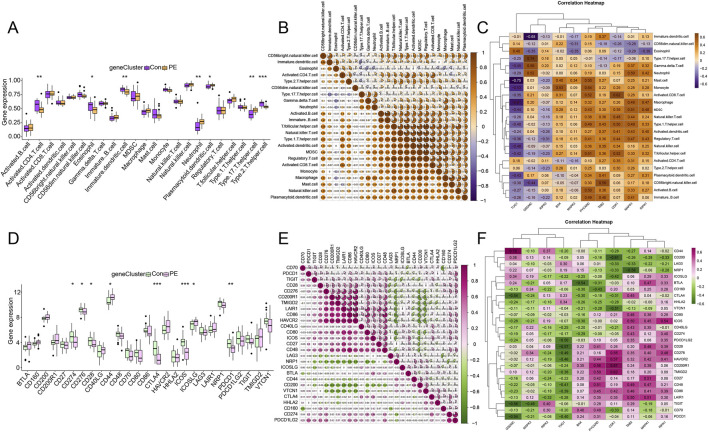
Analysis of the abundance of immune cell and immune checkpoint expression between groups, and the correlation between PANoptosis-related DEGs and immunocytes or immune checkpoints. The boxplots depict different gene expressions of **(A)** immunocytes and **(D)** immune checkpoints in the decidual tissues between PE and normal controls, respectively. * represents *P* < 0.05, ** represents *P* < 0.01, and *** represents *P* < 0.001 compared with normal controls. **(B, E)** The correlation heatmaps between immunocytes and between immune checkpoints, respectively. **(C, F)** The correlation heatmaps between PANoptosis-related DEGs and immunocytes and immune checkpoints, respectively.

Given the known pro-inflammatory characteristics of PANoptosis and the immune-centric nature of decidualization, we next investigated whether the dysregulation of PANoptosis-related genes was associated with alterations in the immune microenvironment of PE decidua. The ssGSEA was performed to identify the quantity of immunocyte infiltration in the decidual tissues of PE. The results demonstrated significant differences in the immune landscape of decidual tissues between the PE and control samples (*P* < 0.05). Specifically, the decidual tissues of PE exhibited an increased proportion of neutrophils, plasmacytoid dendritic cells, and T helper type 17 cells while displaying a decreased level of activated CD4^+^ T cells, eosinophils, immature dendritic cells, and T helper type 2 cells when compared with the healthy controls (Figure 5A). Further correlation analysis on the infiltrating immunocytes in PE identified the strongest synergistic effect between MDSC and regulatory T cells (r = 0.91), T follicular helper cells and regulatory T cells (r = 0.91), followed by regulatory T cells and T helper type 1 cells (r = 0.90) (Figure 5B). Conversely, multiple pairs of immune cells exhibiting a negative correlation were derived, including T helper type 17 cells and immature dendritic cells (r = −0.39), followed by gamma delta T cells and eosinophils (r = −0.36). Furthermore, correlation analysis showed PANoptosis-related DEGs were closely associated with diverse immunocytes that varied in abundance between the groups. Specifically, GSDMC showed the strongest positive correlation with T helper type 17 cells (r = 0.74), and TUG1 exhibited the strongest negative correlation with mast cells (r = −0.75) (Figure 5C).

In addition, the results showed that 8 out of 27 immune checkpoints were differently expressed in the decidual tissues between groups, including CD274, CD276, CD28, CD44, CTLA4, ICOS, ICOSLG, and PDCD1 (Figure 5D). Likewise, the most positive link was discovered between CD86 and HAVCR2 (r = 0.86), while the most negative association was observed between NRP1 and CD86, CD200 and CD200R1, CD200 and LAIR1 (r = −0.62) (Figure 5E). Furthermore, the connections between PANoptosis-related DEGs and immune checkpoints were also identified. Specifically, GSDMC was most positively related to CD44 (r = 0.7), whereas CDK1 showed the most negative correlation with CD200 (r = −0.61) (Figure 5F).

### Construction of a diagnostic model for PE based on PANoptosis-related DEGs by machine learning

**FIGURE 6:**
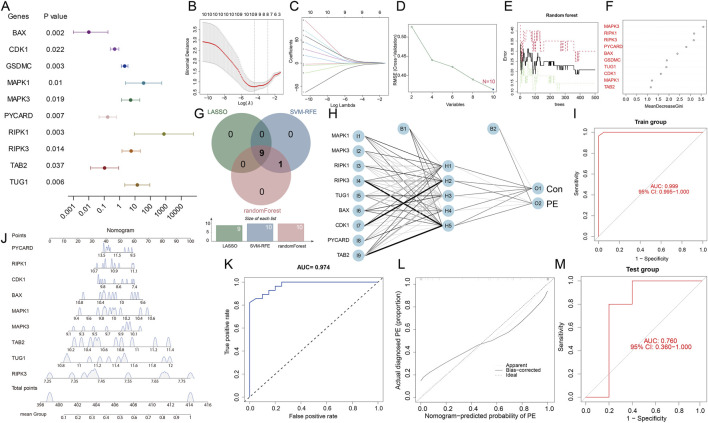
Screening signature genes for PE based on PANoptosis-related DEGs by machine learning. **(A)** BLR on PANoptosis-related DEGs in the decidual tissues between PE and normal controls. **(B, C)** Screening signature genes through the LASSO algorithm in the decidual tissues between PE and normal controls. **(D)** Screening signature genes through the SVM-RFE algorithm in the decidual tissues between PE and normal controls. **(E, F)** Screening signature genes through the RF algorithm and the importance of signature genes by the RF algorithm in the decidual tissues between PE and normal controls. **(G)** The Venn diagrams of intersected genes from these three algorithms. The corresponding names and numbers of genes are noted on figures. **(H)** The artificial neural network model for overlapped genes. I1–I9 are the input layers (the score and weight of 9 signature genes), H1–H5 are the hidden layers, and O1–O2 are the output layers (sample attributes). **(I)** ROC curves for assessing the diagnostic effectiveness of the ANN model in the training set. **(J)** The nomogram prediction model. **(K)** The ROC for evaluating the discriminative performance of the nomogram predictive model. **(L)** The visual calibration plot for the internal validation of the prediction model. **(M)** ROC curves for assessing the diagnostic utility of the artificial neural network model in the testing set composed of decidualized human endometrial stromal cell samples.

Machine learning has been widely applied in disease classification and biomarker discovery, providing valuable tools for exploratory prediction modeling. Firstly, BLR was conducted, and the results revealed that 10 PANoptosis-related DEGs were independent factors (Figure 6A). Then, signature gene screening based on PANoptosis-related DEGs was performed by LASSO, SVM-RFE and RF algorithms to identify candidate biomarkers and construct a predictive model for PE. As shown in Figures 6B,C, nine potential biomarkers were identified via the LASSO regression algorithm, the SVM-RFE and RF algorithms revealed that all ten genes showed potential discriminative value between PE and control samples (Figures 6D–F). To assess the robustness and generalizability of the machine learning models, their classification performance was evaluated on the testing set using ten-fold cross-validation, as summarized in Table 3. Model performance was assessed using sensitivity, specificity, positive predictive value (PPV), negative predictive value (NPV), accuracy, and the area under the receiver operating characteristic curve (AUC). Collectively, these metrics provide a comprehensive evaluation of each model’s predictive performance for PE. Finally, nine signature genes (MAPK3, RIPK1, RIPK3, PYCARD, BAX, TUG1, CDK1, MAPK1, and TAB2) were selected by intersecting the three methods (Figure 6G). Based on these genes, ANN model was constructed (Figure 6H), and ROC curve analysis demonstrated excellent predictive performance with an AUC values of 0.999 (95% CI: 0.995–1.000) (Figure 6I). For better performance in the prediction of PE, the nomogram model was constructed on the above signature genes (Figure 6J). The ROC curve was utilized to ascertain the particularity and sensitivity of each signature gene and the nomogram model in predicting PE. The data revealed a greater AUC value for the nomogram compared to each hub gene, implying that the nomogram exhibited improved predictive performance compared with individual genes within the analyzed dataset (Supplementary Figure 2; Figure 6K). The calibration curves displayed that the predictive value of the established nomogram diagnostic model closely matched the performance of the ideal model (Figure 6L), demonstrating a good predictive value in PE.

**TABLE 3:** The predictive performance of machine learning models.

Model	Sensitivity	Specificity	PPV	NPV	Accuracy	AUC
LASSO	0.75	0.6	0.6	0.75	0.667	0.75
SVM-RFE	0.75	0.6	0.6	0.75	0.667	0.8
Random forest	0.75	0.8	0.75	0.8	0.778	0.9

Then, the signature genes identified through machine learning were further validated in the testing set composed of decidualized human endometrial stromal cells (GSE91077). And the ROC curve analysis suggested that the ANN model retained a certain level of predictive performance in the decidualized human endometrial stromal cell samples (AUC = 0.760, 95% CI: 0.360–1.000) (Figure 6M).

### Consensus clustering analysis

**FIGURE 7:**
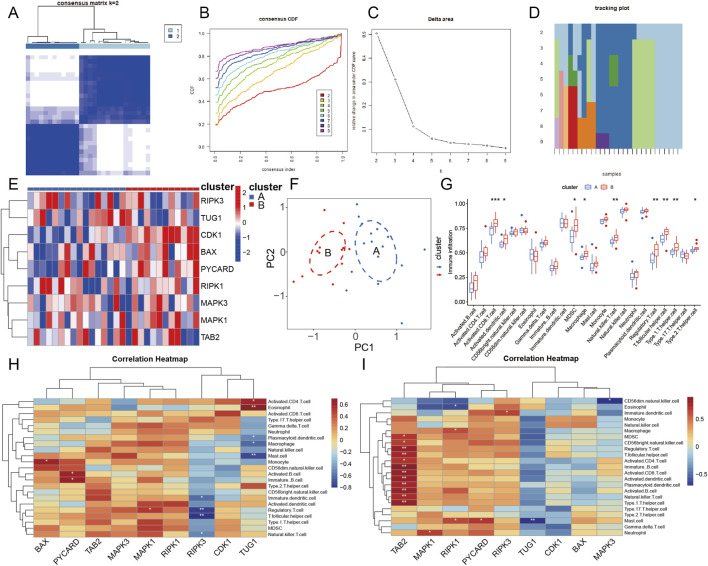
Consensus clustering analysis on PE decidual samples, immune infiltration analysis and correlation analysis on two subtypes. **(A)** The consistency clustering result diagram. **(B)** Consensus among clusters for each category number k. **(C)** Delta area curves for consensus clustering reflecting the relative alteration in area under the CDF curve for each category number k compared to k-1. The horizontal axis represents the category number k and the vertical axis represents the relative change in area under CDF curve. **(D)** The tracking plot of samples. **(E)** The heatmap of the two clusters classified by the 9 signature genes. **(F)** PCA under the PANoptosis-related DEGs modification pattern. **(G)** The boxplot of the immune infiltration levels of immunocytes in the subtypes, with blue indicating the subtype A and red indicating the subtype B. **(H, I)** The correlation heatmaps between PANoptosis-related DEGs and immunocytes in subtype A and subtype B of PE samples, respectively.

In addition, the decidual tissue samples of PE were clustered on the basis of the expression profiles of signature genes via consensus clustering analysis (Figure 7A). PE decidual samples were classified into two subtypes according to a consensus matrix plot, a CDF plot, relative alterations in the area under the CDF curve, and a tracking plot (Figures 7A–D). Heatmap and PCA displayed a remarkable difference between the two subtypes (Figures 7E,F). Subsequently, immune infiltration analysis and correlation analysis were carried out to mine the immune features of different subtypes of PE and identify their association with signature genes (Figures 7G–I). Subtype B showed a higher abundance of immunocytes than subtype A. The correlation analysis demonstrated that the most positive association was between TUG1 and eosinophils (r = 0.697), followed by PYCARD and immature B cells (r = 0.621) in subtype A. Similarly, TAB2 was most positively related to immature B cells (r = 0.909), followed by TAB2 and T helper Type 1 cells (r = 0.846) in subtype B of PE samples.

### Validation of PANoptosis-related signature genes in primary human decidual stromal cells (HDSCs)

**FIGURE 8:**
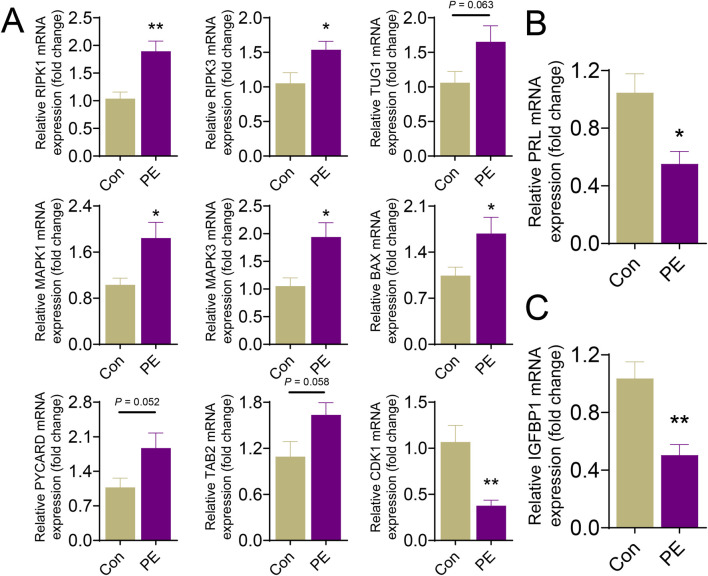
Validation of PANoptosis-related signature genes and decidualization markers in primary human decidual stromal cells (HDSCs) from pre-eclampsia pregnancies. **(A)** qRT-PCR analysis of the mRNA expression of nine machine learning-identified PANoptosis-related signature genes in HDSCs isolated from PE patients and healthy controls. **(B, C)** qRT-PCR analysis of the mRNA expression of decidualization markers **(B)** PRL and **(C)** IGFBP1 in HDSCs. * represents *P* < 0.05 and ** represents *P* < 0.01 compared with the controls.

To further validate the PANoptosis-related signature genes identified by machine learning, primary HDSCs isolated from PE patients and healthy controls were analyzed. qRT-PCR results showed that the mRNA expression of the nine PANoptosis-related signature genes exhibited differential expression patterns in HDSCs derived from PE compared with control cells (Figure 8A). Among these genes, BAX, MAPK1, MAPK3, RIPK1, RIPK3 were markedly upregulated (*P* < 0.05), CDK1 was, markedly downregulated (*P* < 0.05), consistent with the predictions from machine learning analysis (Figure 8A). To assess the functional impact of PANoptosis activation on decidualization, the mRNA levels of decidualization markers were measured. qRT-PCR revealed that the expression levels of PRL and IGFBP1 were significantly reduced in HDSCs from PE samples compared with controls (*P* < 0.05) (Figures 8B,C), indicating impaired decidual function.

### Screening and validating potential drugs for decidualization deficiency in PE

**FIGURE 9:**
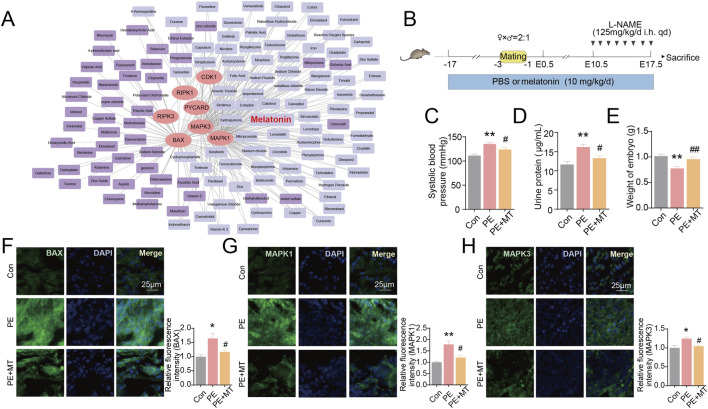
Screening and validating the therapeutic compounds targeting PANoptosis-related signature genes in PE. **(A)** The association network of signature genes with medicine. Core nodes: signature genes. Peripheral nodes: medicine. **(B)** Experimental approach employed for the animal trials. In order to investigate the therapeutic efficacy of melatonin on PE, mice were administered PBS or melatonin as a pre-treatment. The mice were categorized into Con, PE, and PE + MT groups on the basis of whether they received a subcutaneous injection of L-NAME on E10.5. **(C)** The systolic blood pressures (SBPs) of the three groups were measured on E16.5. **(D)** The urine protein levels of the three groups were measured on E16.5. **(E)** The embryo weights of three groups. **(F–H)** Immunofluorescent staining images of BAX, MAPK1, and MAPK3 (green) in the mouse decidual tissue of three groups. Nuclei are counterstained with DAPI (blue). The relative fluorescence intensity of BAX, MAPK1 and MAPK3 in the three groups was quantified, respectively. * represents *P* < 0.05 and ** represents *P* < 0.01 compared with the controls. # represents *P* < 0.05 and ## represents *P* < 0.01 compared with the PE groups. Magnification: Objective lens: ×20, Eyepiece: ×10. Scale bar, 25 μm.

Subsequently, candidate compounds targeting the PANoptosis-related signature genes were further screened. Among these compounds, melatonin was one of the compounds that could target multiple PANoptosis-related signature genes of PE (Figure 9A). Thus, we focused on the effect of melatonin in L-NAME-induced PE model (Figure 9B). As previously reported, a significant increase in systolic blood pressure (*P* < 0.01) and urine protein concentrations (*P* < 0.01) were observed in mice induced by L-NAME (Figures 9C,D), and the embryo weight was notably reduced (*P* < 0.01) (Figure 9E), demonstrating the effective establishment of the PE model. In contrast, melatonin significantly reduced systolic blood pressure (*P* < 0.05) and urine protein levels (*P* < 0.05), and caused a notable increase in embryo weight (*P* < 0.01) (Figures 9C–E). Furthermore, based on the validation results in HDSCs, the PANoptosis-related signature genes (BAX, MAPK1, and MAPK3) were further examined in the decidual tissues of PE mice. And the expressions of BAX, MAPK1, and MAPK3 were increased in PE group (*P* < 0.05), which were significantly reduced by melatonin (*P* < 0.05) (Figures 9F–H). These results suggested that melatonin may modulate the expression of PANoptosis-related signature genes in experimental PE models.

**FIGURE 10:**
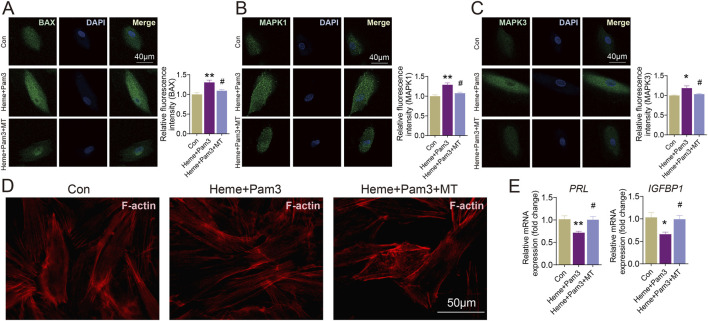
The effect of melatonin on defective decidualization *in vitro* induced by PANoptosis of T-hESCs. **(A–C)** Immunofluorescent staining images of BAX, MAPK1, and MAPK3 (green) in the T-hESCs of three groups. Nuclei are counterstained with DAPI (blue). The relative fluorescence intensity of BAX, MAPK1 and MAPK3 in the three groups was quantified, respectively. **(D)** The level of F-actin of T-hESCs in different groups were detected by immunofluorescence. **(E)** The level of PRL and IGFBP1 of T-hESCs in different groups were detected by qRT-PCR. * represents *P* < 0.05 and ** represents *P* < 0.01 compared with the controls. # represents *P* < 0.05 compared with the Heme + Pam3 groups. Scale bar, 50 μm.

To further validate the effect of melatonin on PANoptosis and decidualization of T-HESCs, we treated T-HESCs with heme and Pam3 during induced decidualization, with or without melatonin treatment. Accordingly, the fluorescence intensity of PANoptosis-related signature genes (BAX, MAPK1, and MAPK3) was apparently increased due to heme and Pam3 exposure (*P* < 0.05) (Figures 10A–C). Meanwhile, the immunofluorescence and qRT-PCR results displayed the spindle distribution of F-actin (Figure 10D) and substantially declined the mRNA level of PRL and IGFBP1 (*P* < 0.05) (Figure 10E), confirming the presence of decidualization deficiency. In contrast, melatonin treatment markedly downregulated the expressions of BAX, MAPK1, and MAPK3 according to immunofluorescence (*P* < 0.05) (Figures 10A–C). Furthermore, melatonin distinctly alleviated spindle change of F-actin and elevated the mRNA levels of PRL (*P* < 0.01) and IGFBP1 (*P* < 0.05) (Figures 10D,E). These results indicated that PANoptosis may be involved in decidualization deficiency, and that melatonin may partially exert its effects through modulation of PANoptosis-related genes.

### MAPK3 knockdown attenuates PANoptosis-associated decidualization deficiency in T-HESCs

**FIGURE 11:**
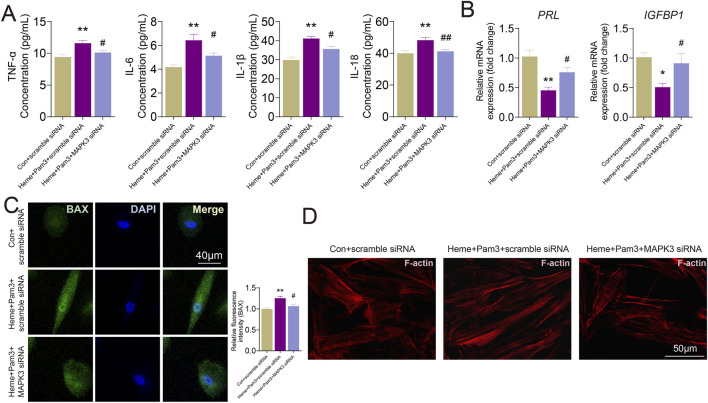
MAPK3 knockdown attenuates PANoptosis-associated decidualization deficiency in T-HESCs. **(A)** ELISA analysis of pro-inflammatory cytokine (TNF-α, IL-6, IL-1β, IL-18) levels in culture supernatants. **(B)** qRT-PCR analysis of decidualization markers PRL and IGFBP1 mRNA levels. **(C, D)** Immunofluorescence staining for **(C)** BAX (green) and **(D)** F-actin (phalloidin, red). * represents *P* < 0.05 and ** represents *P* < 0.01 compared with the controls. # represents *P* < 0.05 compared with the Heme + Pam3 groups. Scale bar, 50 μm.

To further determine whether MAPK3 functionally contributes to PANoptosis-associated decidual dysfunction, MAPK3 was silenced in T-HESCs using siRNA prior to PANoptosis induction. Following decidualization induction and subsequent heme and Pam3 stimulation, MAPK3 knockdown markedly altered the inflammatory and decidualization-related responses. ELISA analysis of culture supernatants showed that PANoptosis induction significantly increased the secretion of pro-inflammatory cytokines, including TNF-α, IL-6, IL-1β, and IL-18, compared with control cells (*P* < 0.01) (Figure 11A). Notably, MAPK3 knockdown substantially reduced the levels of these cytokines under PANoptosis-inducing conditions (*P* < 0.05), indicating an attenuation of PANoptosis-associated inflammatory responses (Figure 11A). Consistent with these findings, qRT-PCR analysis demonstrated that the mRNA levels of the decidualization markers PRL and IGFBP1 were markedly decreased following PANoptosis induction (*P* < 0.05). Silencing MAPK3 significantly restored the mRNA levels of both PRL and IGFBP1 compared with PANoptosis-treated cells transfected with control siRNA (*P* < 0.05), suggesting a partial rescue of decidualization capacity (Figure 11B). Furthermore, immunofluorescence staining revealed that PANoptosis induction led to a pronounced increase in BAX expression accompanied by marked disruption of F-actin organization, characterized by fragmented and disordered actin filaments. In contrast, MAPK3 knockdown significantly reduced BAX fluorescence intensity and partially restored F-actin cytoskeletal integrity under PANoptosis-inducing conditions (Figures 11C,D). Collectively, these results demonstrate that MAPK3 may be involved in mediating PANoptosis-associated inflammatory activation and decidualization impairment in T-HESCs, supporting a critical role for MAPK3 in decidual dysfunction of PE.

## Discussion

### Main findings

This study provides initial evidence suggesting a significant association between PANoptosis and decidualization deficiency in PE. Through comprehensive bioinformatics analysis of decidual tissue, we identified ten PANoptosis-related DEGs (GSDMC, RIPK1, TUG1, MAPK1, RIPK3, MAPK3, BAX, PYCARD, CDK1, TAB2) intricately interacting within a PPI network. Concurrently, we observed profound immune dysregulation in PE decidua, characterized by altered infiltration of various immunocytes correlating with PANoptosis-related genes, especially GSDMC and Th17 cells. Crucially, we developed and internally validated exploratory prediction models (ANN, nomogram) using nine machine learning-selected PANoptosis signature genes (MAPK3, RIPK1, RIPK3, PYCARD, BAX, TUG1, CDK1, MAPK1, TAB2), which enabled the classification of PE samples into two distinct immunological subtypes based on transcriptomic profiles. Importantly, these PANoptosis signature genes were further validated in the primary HDSCs derived from PE patients and healthy controls. In parallel, functional loss-of-function experiments in decidualizing T-HESCs demonstrated that MAPK3 knockdown attenuated PANoptosis-associated inflammatory responses, reduced BAX expression, and partially restored decidualization features, providing support for its involvement in decidual dysfunction. Furthermore, we identified melatonin as a potential therapeutic candidate, demonstrating its efficacy in suppressing the expression of these PANoptosis-related signature genes and improving decidualization in both *in vivo* PE models and *in vitro* T-HESCs.

### Interpretation

Despite extensive research spanning several decades, PE remains a leading cause of maternal and neonatal morbidity and mortality, largely due to its unclear etiology and lack of effective treatments (Dimitriadis et al., 2023). Lately, there has been a growing interest regarding the roles of inflammation and various specific cell death mechanisms in the development of PE (Cheng et al., 2019; Deer et al., 2023; Yu et al., 2023). Interestingly, PANoptosis essentially recruits fundamental elements of several distinct cell death pathways to carry out regulated inflammatory cell death. Consequently, PANoptosis has emerged as a potential target for both diagnosis and therapeutic intervention in PE (Zhu et al., 2023). However, there is still a dearth of conclusive research that states the involvement of PANoptosis in the process of PE development.

Initially, by performing enrichment analysis on the DEGs in the decidual tissue of PE, we could unravel that these genes were predominantly involved in inflammation and various specific cell death, including the mesenchymal cell apoptotic process, the TNF signaling pathway, and the Toll-like receptor signaling pathway. Mesenchymal cells possess the capacity for self-renewal, proliferation, and multilineage differentiation. During the luteal phase and pregnancy, decidualization induces a morphological transformation of stromal cells, shifting them from spindle-shaped to rounder epithelioid cells. This mesenchymal-to-epithelial transition (MET) endows mesenchymal cells with epithelial characteristics (Du et al., 2017). Hyperactive apoptosis of mesenchymal stem cells can compromise their decidualization potential, potentially contributing to PE onset (Gu et al., 2022). In addition to aberrant apoptosis, other forms of programmed cell death due to the overactive immunoinflammation response have also been verified to be associated with PE (Lu and Hu, 2019). The TNF signaling pathway has been reported to be overactive in the decidual tissue of PE (Haider and Knöfler, 2009), which may initiate cell pyroptosis along with an inflammatory cascade and induce impaired decidualization (Inoue et al., 1994; Patel et al., 2023). Besides, Toll-like receptor (TLR), belonging to the innate immunity receptor (Santos-Sierra, 2021), has been found to be continuously activated in the decidual tissues and trophoblasts of PE. Specifically, highly expressed TLR can result in extensive inflammatory cell death at the maternal-fetal interface by recruiting immunocytes into the decidual tissue (Afkham et al., 2019; Gierman et al., 2021). Importantly, PANoptosis represents a unique inflammation-mediated cell death mechanism that orchestrates the crosstalk between necroptosis, apoptosis, and pyroptosis (Pandian and Kanneganti, 2022). And PANoptosis can be triggered by the TLR and TNF signaling pathways that the DEGs of PE were enriched in Wang and Kanneganti (2021) and Sundaram et al., (2023). Moreover, various pathological features of PE, including oxidative stress, ischemia, and hypoxia, are closely linked to PANoptosis (Liu et al., 2023; She et al., 2023). These processes surpass the maternal elimination capability, which in turn compromises the decidualization process and exacerbates further cell apoptosis and necrosis within the maternal-fetal interface, thus eventually contributing to different severity of PE (Feinberg, 2006; Yu et al., 2019). Collectively, these findings support a potential association between PANoptosis and decidual dysfunction in PE.

Broadly speaking, decidualization is a highly coordinated process that requires not only stromal cell differentiation but also the recruitment and fine-tuning of local immune responses to establish and maintain maternal–fetal tolerance (Meng et al., 2023). In this context, we examined the immune landscape of decidual tissues in PE. Our immune infiltration analysis revealed aberrant changes in multiple immune cell populations in PE decidua, such as neutrophils, plasmacytoid dendritic cells, T helper type 17 cells, activated CD4^+^ T cells, eosinophils, immature dendritic cells, and T helper type 2 cells. Taking T helper (Th) cells as an example, Th 2 and Th 17 are both vital subsets of CD4^+^ T cells (Solano, 2019). Th2 dominance at the decidua is critical for immune tolerance during normal pregnancy (Michimata et al., 2002), whereas a reduction in Th2 activity in PE has been associated with a shift toward a pro-inflammatory immune milieu (Jianjun et al., 2010; Meng et al., 2023). Such immune imbalance may contribute to excessive inflammatory signaling, destroying the decidual extracellular matrix, and interrupting regular EVT invasion (Wang et al., 2020). In addition, existing data has demonstrated a significant elevation in Th17 cells in the decidua of PE (Wu et al., 2014). Elevated Th17 activity and IL-17 production may amplify local inflammation through the induction of cytokines and chemokines, such as IL-6, TNF-α, G-CSF, CXCL1, and acute-phase proteins, thereby aggravating tissue infiltration and injury (Wang et al., 2010; Hosseini et al., 2018). Given the established links between PANoptosis and inflammatory signaling, we further explored the correlation between PANoptosis-related DEGs and immune cell infiltration patterns. Notably, the GSDMC demonstrates the strongest positive association with Th17 cells. GSDMC, a member of the gasdermin family, has been implicated in pyroptosis-mediated inflammatory responses (Zhang et al., 2021). Previous studies suggested that Th17 cells may stimulate the secretion of GSDMC by secreting IFN-γ, which subsequently increases the cytotoxicity of T cells (Cosmi et al., 2014; Chao et al., 2023; Wang et al., 2024). Collectively, our data suggest that altered immune homeostasis in PE decidua may coexist with PANoptosis-related molecular dysregulation and together contribute to decidualization deficiency. Future studies will be required to elucidate the precise molecular mechanisms linking PANoptosis, immune dysregulation, and decidualization deficiency in PE.

Considering diagnosis, conventional methods for PE diagnosis depend on evaluating clinical and laboratory indicators, including new-onset hypertension, proteinuria, pre-existing renal conditions, and uteroplacental lesions or limited fetal growth during the early stages of pregnancy (Mol et al., 2016). However, due to the unclear etiology, complexity of risk factors, and probable existence of numerous pathogenic phenotypes linked to PE, the rates of identifying PE are poor (Deer et al., 2023; Dimitriadis et al., 2023). The combination of transcriptome microarray-based bioinformatics and machine learning provides a potent strategy to overcome this obstacle, improving the performance of exploratory prediction models. Here, nine signature PANoptosis-related genes (MAPK3, RIPK1, RIPK3, PYCARD, BAX, TUG1, CDK1, MAPK1, and TAB2) with potential discriminative relevance for PE were identified by utilizing LASSO, SVM-RFE, and RF algorithms. Additionally, based on the above signature genes, ANN and nomogram diagnostic models for PE were established and further validated in the testing sets, which supported the predictive performance of these PANoptosis-related genes. Importantly, to strengthen the translational relevance of our bioinformatics findings, we further validated the PANoptosis-related signature genes in the primary HDSCs derived from PE patients and healthy controls. On the whole, the expression trends of the nine signature genes in HDSCs of PE corresponded to the machine-learning predictions, as exemplified by the pronounced upregulation of BAX, MAPK1, and MAPK3. Moreover, the concomitant reduction of decidualization markers (PRL and IGFBP1) observed in PE decidua suggest a close association between PANoptosis-related transcriptional dysregulation and impaired decidual function. Collectively, these findings provide biological validation at the human tissue level for the identified PANoptosis-related signature genes and support their potential involvement in decidualization deficiency during PE. Furthermore, consensus clustering analysis revealed the existence of two different subtypes of PE on the basis of the expression profiles of the nine PANoptosis-related signature genes. Analysis of immune infiltration revealed that subtype B exhibited a markedly elevated level of immunocytic infiltration compared to subtype A. Besides, these candidate diagnostic genes were found to be substantially related to the immunocyte infiltration in PE, indicating their potential role in distinguishing distinct immunological subtypes of PE and impacting PE through interactions with immune inflammation pathways. To conclude, these findings highlight the potential predictive relevance of PANoptosis-related genes connected to PANoptosis. Also, our research led to a profound comprehension of PANoptosis in the pathogenesis of decidualization deficiency in PE and holds the potential to lay a foundation for future studies aimed at identifying more sensitive candidate biomarkers. However, further prospective studies and large, multi-center external validation cohorts will be required to confirm the generalizability of these findings and to determine the clinical relevance of PANoptosis-related signatures beyond the current exploratory analyses.

Although transcriptional profiling and validation in human decidual tissues provided strong biological relevance for PANoptosis-related signature genes in PE, such correlative evidence alone is insufficient to establish a causal role for individual genes in decidual dysfunction. Among the identified PANoptosis-related signature genes, MAPK3 was selected for further functional investigation based on its central role in integrating inflammatory and stress-related signaling pathways. As a core component of the ERK1/2 pathway, MAPK3 is activated downstream of Ras/Raf/MEK signaling and regulates diverse cellular processes, including proliferation, differentiation, apoptosis, and inflammation (Roskoski, 2012; Lee et al., 2013). Previous studies have suggested that precise temporal regulation of ERK1/2 signaling is required for normal decidualization, whereas dysregulated ERK activity under inflammatory or stress conditions may compromise stromal cell differentiation (Lee et al., 2013). Importantly, independent functional experiments in our study further supported the pathogenic relevance of MAPK3 in PANoptosis-associated decidual dysfunction. Specifically, our results demonstrated that siRNA-mediated MAPK3 knockdown markedly attenuated PANoptosis-associated inflammatory responses, as evidenced by reduced secretion of pro-inflammatory cytokines, including IL-1β, IL-6, TNF-α, and IL-18. In parallel, MAPK3 silencing significantly decreased the expression of BAX and partially restored the F-actin organization and the expression of decidualization markers PRL and IGFBP1 under PANoptosis-inducing conditions. Together, these findings suggest that MAPK3 may represent a key component of PANoptosis-associated signaling pathways implicated in decidualization deficiency in PE.

Currently, the management of PE primarily involves lifestyle interventions, careful monitoring, antihypertensive therapy, and low-dose aspirin, all of which aim to prolong pregnancy rather than cure the disease (Mol et al., 2016). In this regard, it is imperative to promptly investigate the potential pharmaceuticals. Here, through the CTD and Drugbank databases, the candidate compounds that potentially targeted PANoptosis-related signature genes were screened. Melatonin, a potent antioxidant, plays a pivotal role in female reproduction through both receptor-mediated and receptor-independent antioxidant mechanisms (de Almeida Chuffa et al., 2019; Cui et al., 2021). Relevant clinical research has offered robust evidence indicating a correlation between diminished melatonin levels and the progression of PE (Zeng et al., 2016), and melatonin administration has been reported to exhibit antihypertensive effects in PE patients (Hobson et al., 2018). Consistently, our findings indicate that therapeutic melatonin treatment (10 mg/kg/day) significantly lowered systolic blood pressure and reduced 24-h urine albumin excretion in the L-NAME-induced PE murine model. In addition, as previously reported, melatonin administration could significantly improve decidualization deficiency by elevating the expression profile of PRA and p53, in which PRA could influence the decidual response and luminal cell differentiation, and p53 can regulate the uterus through leukemia inhibitory factor (LIF), thus improving the number of implantation sites and the litter size in the pregnant murine model (He et al., 2015). Nevertheless, the available evidence regarding the impact of melatonin on improving decidualization deficiency in PE remains inadequate. Extending these findings, we observed that melatonin markedly inhibited the expression of PANoptosis-related signature genes both in decidual tissue of the PE murine model and in decidualizing T-HESCs treated with heme and Pam3. These results were in conjunction with previous research reporting melatonin-mediated modulation of BAX, MAPK1, and MAPK3 (Lin et al., 2017; Maria et al., 2017; Xu et al., 2020; Diao et al., 2023). Furthermore, we discovered that under the treatment with melatonin, decidualization was significantly improved in parallel with the decrease in PANoptosis-related signature genes. Collectively, melatonin may represent a potential therapeutic candidate for modulating PANoptosis and alleviating decidualization deficiency in PE. Nevertheless, additional mechanistic studies and independent validation in larger animal models and clinical cohorts are necessary before its clinical applicability can be considered.

### Strengths and limitations

In this study, we demonstrated for the first time a close association between PANoptosis and decidualization deficiency in PE. Our findings not only provide novel insights into the role of PANoptosis in the pathogenesis of PE but also identify potential biomarkers for PE diagnosis. Furthermore, we highlighted that compounds targeting PANoptosis-related signature genes, such as melatonin, may have therapeutic potential, which was preliminarily validated in both *in vivo* and *in vitro* models.

Nevertheless, several limitations should be acknowledged. First, although the combination of bioinformatics analyses and experimental validation offers initial evidence for the involvement of PANoptosis in decidualization deficiency, the underlying molecular mechanisms driving PANoptosis activation in PE remain to be elucidated. Second, while the signature genes identified in this study demonstrated strong diagnostic performance, further research is needed to fully assess their specificity, clinical applicability, and potential integration into diagnostic frameworks. Third, despite employing multiple independent datasets and experimental validations, the sample sizes were relatively limited. Future large-scale, multi-center studies are required to further validate the robustness and generalizability of the identified PANoptosis-related signatures. Finally, although candidate compounds targeting PANoptosis were screened through public databases and partially validated in experimental models, further preclinical and clinical investigations are required to determine their safety, efficacy, and optimal dosing for PE therapy.

## Conclusion

To sum up, this study utilized bioinformatics analysis to mine the potential involvement of PANoptosis in decidual dysfunction in PE. Besides, this study revealed a potential association between PANoptosis and the abnormal immune infiltration landscape in PE. In particular, a collaborative strategy of machine learning and external databases evaluated the potential of nine PANoptosis-related genes as candidate biomarkers with potential predictive relevance for PE, including MAPK3, RIPK1, RIPK3, PYCARD, BAX, TUG1, CDK1, MAPK1, and TAB2. Importantly, these PANoptosis signature genes were further validated in the HDSCs derived from PE patients and healthy controls. Importantly, functional loss-of-function experiments provided support for the involvement of MAPK3 in PANoptosis-associated decidual dysfunction, as MAPK3 knockdown attenuated inflammatory responses, reduced BAX expression, and partially restored decidualization features. Moreover, through the *in vivo* and *in vitro* models, the potential therapeutic value of melatonin in PE by inhibiting PANoptosis activation and decidualization deficiency was explored and supported in in vivo and *in vitro* models. In this way, the study provides us with a deeper comprehension of the associations between PANoptosis and PE and offers a basis for future research into diagnostic and therapeutic strategies for PE.

## Data Availability

The original contributions presented in the study are included in the article/Supplementary Material, further inquiries can be directed to the corresponding authors.
