# Supplementary material for: Leveraging PANoptosis-associated genes for unraveling implication of decidualization deficiency in pre-eclampsia via transcriptome data and experiment validation
Source: Front Cell Dev Biol. 2026 Mar 4;14:1677798. doi: 10.3389/fcell.2026.1677798 (PMC12996107; doi:10.3389/fcell.2026.1677798)
Supplement: Supplementary file 2 [file Table1.docx]

Supplementary Table 1. Sequences of primers.

| Primers | Forward | Reverse |
| --- | --- | --- |
| MAPK3-human | CTCTGCCCTCCAAGACCAAG | CCGTCGGGTCATAGTACTGC |
| RIPK1-human | CCTGGAGAGTGCAGAACTGG | CGGCTGTGTCTCAGTCTGTT |
| RIPK3-human | TGGCCCCAGAACTGTTTGTT | GGTTCGGTTGGCAACTCAAC |
| PYCARD-human | GGTCACAAACGTTGAGTGGC | AGAGCTTCCGCATCTTGCTT |
| BAX-human | AAGGTGCCGGAACTGATCAG | GTCTTGGATCCAGCCCAACA |
| TUG1-human | TGAGCAAGCACTACCACCAG | ACTCAGCAATCAGGAGGCAC |
| CDK1-human | CTAGGGCAGAGTGGTGGTTG | CCTCTGCAGAGTGGTTTGGT |
| MAPK1-human | TAGTGACACGGAACAGCACC | TCCATCCCACTGGCTTGTTC |
| TAB2-human | CCCTGAACGTGTCTCCACTC | TGGGGTATTTCGCTGCACAT |
| PRL-Human | CAAGGAGCAAGCCCAACAGATG | TTCCGTGACCAGATGATACAGAGG |
| IGFBP1-Human | CGCCACCAGCCCAGAGAG | ACAGTCAGCAGGAGCAGTACC |
