# Supplementary figures and images for: Leveraging PANoptosis-associated genes for unraveling implication of decidualization deficiency in pre-eclampsia via transcriptome data and experiment validation
Source: Front Cell Dev Biol. 2026 Mar 4;14:1677798. doi: 10.3389/fcell.2026.1677798 (PMC12996107; doi:10.3389/fcell.2026.1677798)

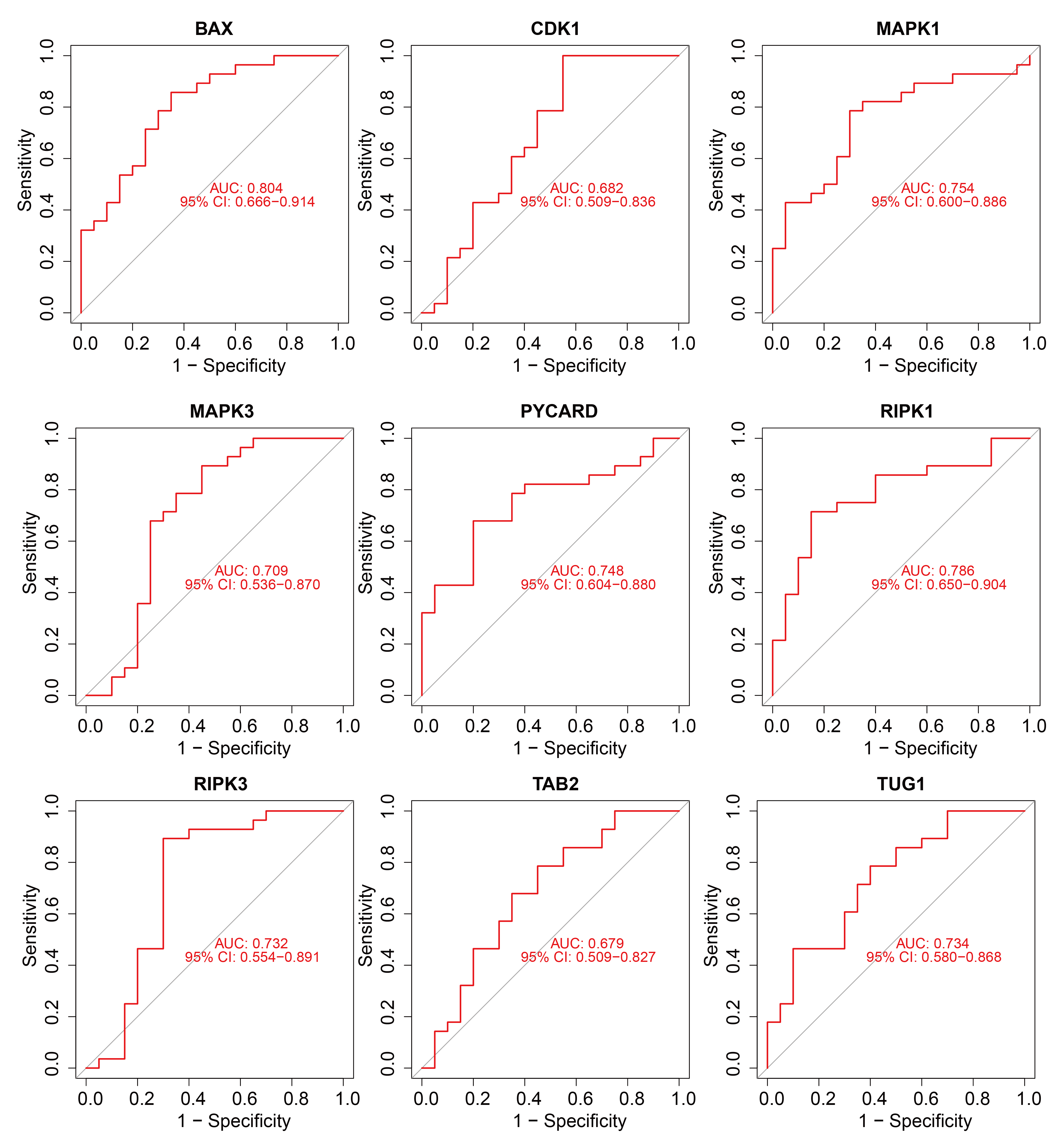

Supplement: Supplementary file 3 [file Image2.tif]

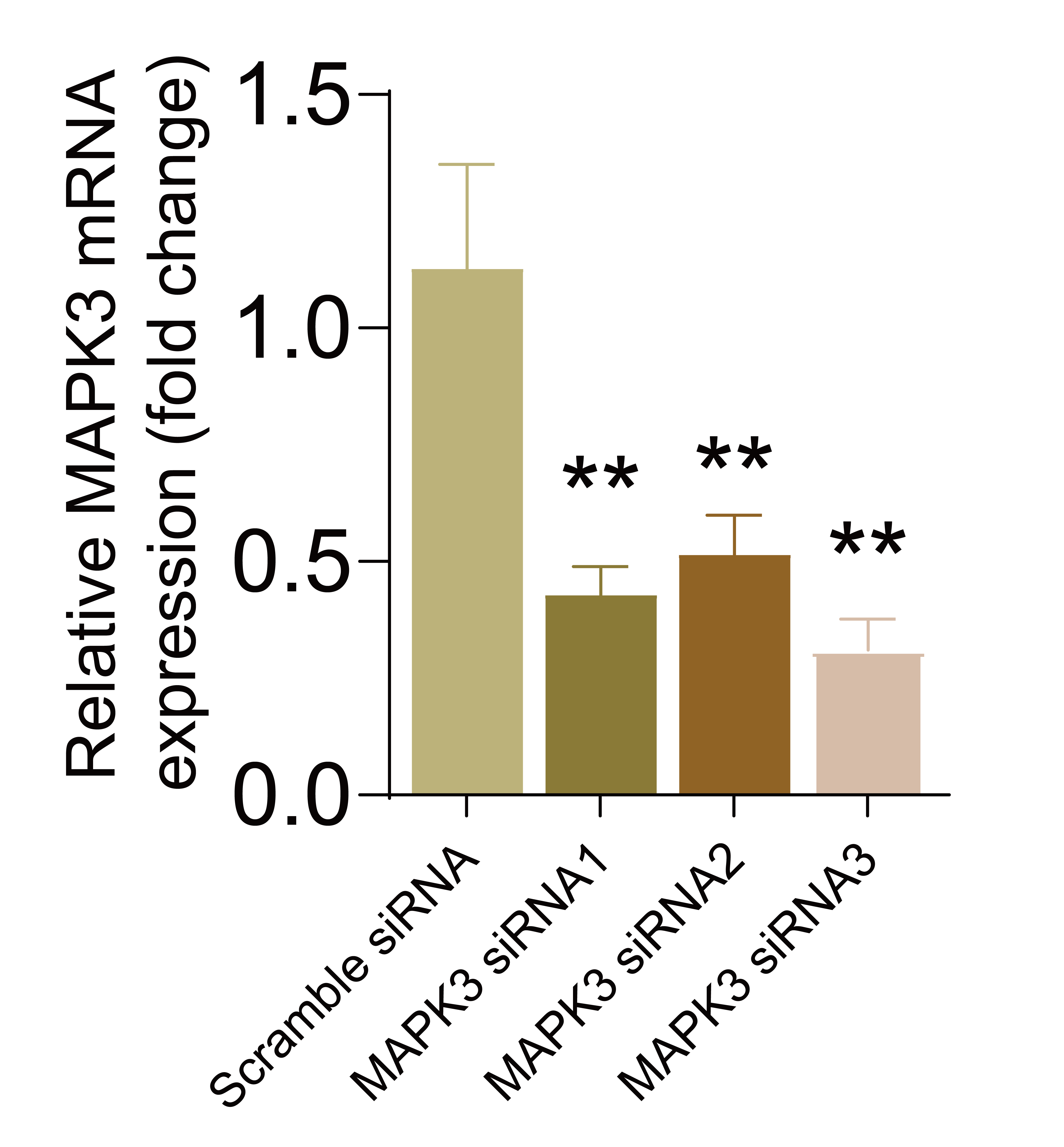

Supplement: Supplementary file 4 [file Image1.tif]
